# A Novel Angiopoietin-2 Selective Fully Human Antibody with Potent Anti-Tumoral and Anti-Angiogenic Efficacy and Superior Side Effect Profile Compared to Pan-Angiopoietin-1/-2 Inhibitors

**DOI:** 10.1371/journal.pone.0054923

**Published:** 2013-02-06

**Authors:** Markus Thomas, Yvonne Kienast, Werner Scheuer, Monika Bähner, Klaus Kaluza, Christian Gassner, Frank Herting, Ulrich Brinkmann, Stefan Seeber, Anita Kavlie, Martin Welschof, Stefan Ries, K. Michael Weidner, Jörg T. Regula, Christian Klein

**Affiliations:** 1 Discovery Oncology, Pharma Research and Early Development, Roche Diagnostics GmbH, Penzberg, Germany; 2 Biologics Research, Pharma Research and Early Development, Roche Diagnostics GmbH, Penzberg, Germany; 3 Affitech Research AS, Oslo, Norway; King's College London, United Kingdom

## Abstract

There is increasing experimental evidence for an important role of Angiopoietin-2 (Ang-2) in tumor angiogenesis and progression. In addition, Ang-2 is up-regulated in many cancer types and correlated with poor prognosis. To investigate the functional role of Ang-2 inhibition in tumor development and progression, we generated novel fully human antibodies that neutralize specifically the binding of Ang-2 to its receptor Tie2. The selected antibodies LC06 and LC08 recognize both rodent and human Ang-2 with high affinity, but LC06 shows a higher selectivity for Ang-2 over Ang-1 compared to LC08 which can be considered an Ang-2/Ang-1 cross-reactive antibody. Our data demonstrate that Ang-2 blockade results in potent tumor growth inhibition and pronounced tumor necrosis in subcutaneous and orthotopic tumor models. These effects are attended with a reduction of intratumoral microvessel density and tumor vessels characterized by fewer branches and increased pericyte coverage. Furthermore, anti-Ang-2 treatment strongly inhibits the dissemination of tumor cells to the lungs. Interestingly, in contrast to the Ang-2/Ang-1 cross-reactive antibody LC08 that leads to a regression of physiological vessels in the mouse trachea, the inhibition with the selective anti-Ang-2 antibody LC06 appears to be largely restricted to tumor vasculature without obvious effects on normal vasculature. Taken together, these data provide strong evidence for the selective Ang-2 antibody LC06 as promising new therapeutic agent for the treatment of various cancers.

## Introduction

Anti-angiogenesis has emerged in the last few years as an effective therapy to target the tumor stromal compartment [1] and is thought to act in a broader fashion compared to cytotoxic therapies.

Angiopoietin-1 (Ang-1) and Angiopoietin-2 (Ang-2) are functional ligands of the Tie2 receptor tyrosine kinase that is expressed on endothelial cells [2]–[4]. Ang-1 is expressed by pericytes, smooth muscle cells and fibroblasts and acts in a paracrine manner as a physiological agonist of Tie2. It acts as a maturation factor that stabilizes the mature vasculature by promoting recruitment of pericytes and smooth muscle cells [5]. In contrast, Ang-2 is expressed by endothelial cells and stored in Weibel-Palade-bodies. It acts as an antagonist of Tie2 by blocking Ang-1 dependent Tie2 activation. However, Ang-2 is also able to context-dependently induce receptor phosphorylation depending on the cell type, cell confluence, stimulation time, or ligand dosage [2], [3], [6]–[9]. Ang-2 is further described to act as a functional destabilization factor, rendering vasculature in a more plastic state amenable to sprouting (under the influence of other angiogenic cytokines such as VEGF) and is found to be particularly increased in highly vascularized tumors and in pro-angiogenic diseases (e.g. macula degeneration, rheumatoid arthritis, osteoarthritis, psoriasis) [10]. Ang-2 is primarily expressed in endothelial vasculature and only rarely in tumor cells [3], [6]. In tumors of different histological origin (e.g., gastric, colon, prostate, breast, AML and brain carcinomas) a shift in the Ang-1-to-Ang-2 expression ratio in favor of Ang-2 was found to be associated with tumor angiogenesis and poor prognosis [11]–[13]. Ang-2 therefore is considered as a major player of the angiogenic switch in the course of tumor progression. In contrast to its broad expression in the vasculature of human tumors, Ang-2 shows limited postnatal expression in normal tissue (e.g. at sites of vascular remodeling like ovary, placenta, uterus) making it a tumor specific target for anti-angiogenic therapies.

Recently, different approaches have been described to target the Angiopoietin axis including Tie2-kinase inhibitors, Fc-fusion proteins and monoclonal antibodies with different specificities that are presently in pre-clinical or clinical stage [14], [15]. AMG386 is a peptibody that targets both Ang-1 and Ang-2 and is currently being evaluated in phase III clinical trials. CVX-060, a highly Ang-2 selective trap molecule (CovX-Body) as well as MEDI3617, a fully human antibody selective for Ang-2, have both recently entered phase I clinical testing [16]–[18]. The most common side effects of these agents include peripheral edema, fatigue, insomnia, upper abdominal pain, back pain, proteinuria, and nausea for AMG386 [19], and fatigue and proteinuria for CVX-060.

As the functional consequences of inhibiting Angiopoietin-2 and -1 are still controversial and poorly understood, we generated fully human antibodies for human and murine Ang-2 and tested their effects in various preclinical models including xenografts (Colo205, KPL4) and angiogenesis models. Systemic treatment of tumor-bearing mice with Ang-2 antibodies resulted in potent tumor growth inhibition and tumor necrosis concomitant with a structural and functional remodeling of tumor vasculature. Moreover, our findings also indicate that Ang-2 inhibition counteracts the dissemination of tumor cells to the lungs. Using the Ang-2 selective anti-Ang-2 antibody LC06, we explored a potential impact on normal quiescent vasculature and found no obvious effects on physiological vessels, whereas treatment with the less Ang-2 selective inhibitory antibody LC08, led to the regression of healthy vessels in the mouse trachea, indicating potential increased toxicity. Taken together, these results suggest that the Ang-2 selective Ang-2 antibody LC06 represents an effective anti-angiogenic therapeutic drug candidate and, due to its possible role in inhibiting angiogenic escape together with a favorable side effect profile may also serve as promising combination partner to complement existing anti-angiogenic therapy regimens for treating patients with solid and hematological malignancies.

## Materials and Methods

### Antibody generation

Binding domains of antibodies against Ang-2 were obtained from human antibody libraries and the obtained V_H_ and V_L_ regions were maturated and then fused to the constant part of an IgG1 antibody. The desired genes were generated by gene synthesis (GeneArt, Regensburg, Germany) and cloned after PCR amplification in suitable expression vectors. Variants of expression plasmids for transient expression in the HEK293-F system (Invitrogen) were applied for the expression of the described antibodies.

Antibodies were purified from filtered cell culture supernatants referring to standard protocols. In brief, antibodies were applied to a Protein A Sepharose column (GE Healthcare) and washed with PBS. Elution of antibodies was achieved at acidic pH followed by immediate neutralization of the sample. Aggregated protein was separated from monomeric antibodies by size exclusion chromatography (Superdex 200, GE Healthcare) in 20 mM Histidine, 140 mM NaCl pH 6.0. Monomeric antibody fractions were pooled, concentrated if required using e.g. a MILLIPORE Amicon Ultra (30 MWCO) centrifugal concentrator and stored at −80°C. Part of the samples were provided for subsequent protein analytics and analytical characterization e.g. by SDS-PAGE, size exclusion chromatography, mass spectrometry and Endotoxin determination.

### Functional characterization of Ang-2 specific antibodies: Binding of antibodies to human Angiopoietin-1/2

ELISA-plates were coated with anti-His⋅Tag® monoclonal antibody (Merck4Biosciences, Germany, Cat.No.70796) for at least 2 hours. The wells were blocked with PBS-T/2%BSA for 1 hour. The antigens hAng-2 (R&D Systems, UK, Cat.No. 623-AN) or hAng-1 (R&D Systems, UK, Cat.No. 923-AN), both proteins with C-terminal His-tag, were captured on plate for 1 hour at room temperature. Plates were washed and dilutions of purified antibodies in PBS were incubated for 1 hour at room temperature. Binding of antibodies was detected with HRP conjugated anti human IgG (GE Healthcare, UK, Cat.No. NA933). After a final wash the plates were incubated with HRP substrate. Absorbance was measured at 405 nm on an EnVision plate reader. EC50 curve fit analysis was performed using XLfit4 analysis plug-in for Excel (model 205).

### Ang-2 SPR (surface plasmon resonance)

The antibody of interest was immobilized on the surface of a C1 SPR Chip. Fixed concentrations of hAng2 or hAng1 (multimeric) were preincubated together with increasing concentrations of the corresponding (immobilized) antibody and injected onto the flowcells. The K_D_ was calculated by plotting the concentrations of free hAng-2 or hAng-1 against the antibody concentrations (affinity in solution).

### Generation of HEK293-Tie2 cell line

In order to determine the interference of Ang-2 antibodies with Ang-2 stimulated Tie2 phosphorylation and binding of Ang-2 to Tie2 on cells, a recombinant HEK293-Tie2 cell line was generated. Briefly, a plasmid coding for full-length human Tie2 was transfected into HEK293 cells (ATCC) and resistant cells were selected in DMEM 10% FCS, 500 µg/ml G418 (Geniticin). Individual clones were isolated, and subsequently analyzed for Tie2 expression by FACS. Clone 22 was identified having high and stable Tie2 expression even in the absence of G418 and subsequently used for cellular assays.

### Ang-2 ligand competition FACS

hAng-2 binding to hTie2 on the cell surface of HEK293-Tie2 was detected via a biotinylated antibody specific for human Ang-2 that does not interfere with the Tie2-Ang-2 interaction and detected with PE-labeled Streptavidin. Titration of Ang-2 antibodies in concentrations ranging from 0.0025 µg/mL up to 10 µg/mL resulted in a dose response curve enabling the determination of EC_50_ values.

### Ang-2 induced Tie2 phosphorylation assay

Inhibition of Ang-2 induced Tie2 phosphorylation by Ang-2 antibodies was measured according to the following assay principle. HEK293-Tie2 clone22 was stimulated with Ang-2 for 5 minutes in the absence or presence of Ang-2 antibody and P-Tie2 was quantified by a sandwich ELISA. Briefly, 2×10^5^ HEK293-Tie2 clone 22 cells per well were grown over night on a Poly-D-Lysine coated 96 well-microtiter plate in DMEM, 10% FCS, 500 µg/ml G418. The next day a titration row of Ang-2 antibodies was prepared in a microtiter and mixed with an Ang-2 (R&D systems # 623-AN) dilution (3.2 µg/ml as 4-fold concentrated solution). Antibodies and Ang-2 were pre-incubated for 15 min at room temperature. The mix was added to the HEK293-Tie2 clone 22 cells (pre-incubated for 5 min with 1 mM NaV_3_O_4_, Sigma #S6508) and incubated for 5 min at 37°C. Subsequently, cells were washed with 200 µl ice-cold PBS +1 mM NaV_3_O_4_ per well and lysed by addition of lysis buffer (20 mM Tris, pH 8.0, 137 mM NaCl, 1% NP-40, 10% glycerol, 2 mM EDTA, 1 mM NaV_3_O_4_, 1 mM PMSF and 10 µg/ml Aprotinin) on ice. After 30 min at 4°C on a microtiter plate shaker lysates were transferred directly into a p-Tie2 ELISA microtiter plate (R&D Systems, R&D #DY990). P-Tie2 amounts were quantified according to the manufacturer's instructions and IC50 values for inhibition were determined using XLfit4 analysis plug-in for Excel (dose-response one site, model 205). IC50 values can be compared within one experiment but might vary from experiment to experiment.

### Cell lines and culture conditions

Colo205 human colorectal cancer cells were originally obtained from ATCC and after expansion deposited in the Roche Penzberg internal cell bank. KPL-4 cells were kindly provided by Professor J. Kurebayashi (Kawasaki Medical School, Kurashiki, Japan). The cell line was established in J. Kurebayashi's lab from the malignant pleural effusion of a breast cancer patient with an inflammatory skin metastasis [20]. Both tumor cell lines were routinely cultured in RPMI 1640 medium (PAA, Laboratories, Austria) supplemented with 10% fetal bovine serum (PAA Laboratories, Austria) and 2 mM L-glutamine, at 37°C in a water-saturated atmosphere at 5% CO_2_ Passages 2–5 were used for transplantation.

### Animals

Female SCID and Balb/c beige mice; age 4–5 weeks at arrival (purchased from Charles River Germany) were maintained under specific-pathogen-free condition with daily cycles of 12 h light/12 h darkness according to committed guidelines (GV-Solas; Felasa; TierschG). All experimental study protocols were reviewed and approved by local government (AZ 55.2-1-54-2531.2-26-09 and AZ 55.2-1-54-2531.2-3-08). Mice were handled according to committed guidelines (GV-Solas; Felasa; TierschG) and animal facility has been accredited by AALAAC.

### Tumor cell injection

At day of injection tumor cells were centrifuged, washed once and resuspended in PBS. After an additional washing with PBS, cell concentration and cell size were determined using a cell counter and analyzer system (Vi-CELL, Beckman Coulter). For injection of Colo205 cells, the final titer was adjusted to 2.5×10^7^ cells/ml, viability >90%. Subsequently, 100 µl of this suspension corresponding to 2.5×10^6^ cells per animal was injected s.c. into the right flank of the mice. Treatment of animals started at day of randomization, at a mean tumor volume of 100 mm^3^. For KPL-4 xenografts, 1.5×10^8^ KPL-4 cells per ml were injected orthotopically at a volume of 20 µl into the right penultimate inguinal mammary fat pad of each anesthetized mouse using a Hamilton microliter syringe and a 30Gx1/2” needle. Treatment of animals started at day of randomization, at a mean tumor volume of 90 mm^3^. Both antibodies were administered at 10 mg/kg once weekly i.p. x 4. Pre-experiments determined that a dose of 10 mg/kg resulted in a maximal anti-tumor effect. The studies were terminated if control animals matched the termination criteria of ulcerated tumor sizes around 1000 mm^3^ (Colo205) and 600 mm^3^ (KPL-4).

### Assessment of disseminated tumor cells by quantification of human DNA

Genomic DNA was isolated using the High Pure PCR Template Preparation kit (Roche Diagnostics GmbH) and quantified using the PicoGreen Quantification kit (Molecular Probes). Primers for human Alu repeats were synthesized by TIB MOLBIOL GmbH Germany and quantitative PCR (qPCR) was performed using the Light-Cycler System (Roche Diagnostics GmbH), as previously described [21].

### Immunohistochemistry

The tumor vasculature was detected in 2-μm paraffin sections by rat anti-mouse CD34 staining (Hycult Biotechnology, Uden, Netherlands) and a biotinylated rabbit anti rat IgG (Vector Labs, Burlingame, CA, USA) or in 10-µm cryosections by rat anti-mouse CD31 staining (BD Pharmingen, San Diego, CA, USA) and a goat anti-rat IgG conjugated with Alexa 546 (Invitrogen, Carlsbad, CA, USA). Vessels were quantified and microvessel density was calculated as vessels per mm^2^ viable tissue. Necrotic areas were calculated as the percentage of necrotic regions (determined by H&E staining) compared to the total tumor area. Areas (necrotic and total) were quantified using CellProfiler software (http://www.cellprofiler.org/). Whole slides of each paraffin section (five tumors per group) were analyzed.

Tumor pericytes were detected either by immunostaining on FFPET with a rabbit anti-desmin polyclonal antibody (Abcam, Cambridge, UK) and a goat anti-rabbit IgG GAR Cy3 antibody (Invitrogen, Carlsbad, CA) as secondary antibody or in 10-μm cryosections by a rabbit anti-mouse polyclonal NG2 antibody (Millipore, Tamecula, CA, USA) detected by goat anti-rabbit Alexa 488 (Invitrogen, Carlsbad, CA, USA). Five to eight random fields in each cryosection (three sections per tumor, five to six tumors per group) were analyzed. Vessel coverage was calculated as the percentage of desmin-positive vessels compared with the number of CD34-positive vessels. Perfused vessels were visualized by i.v. injection of TRITC-lectin (red). Lectin-perfused vessels appear yellow after staining with rat anti-CD34 and Alexa 488 (green)-conjugated goat-anti-rat antibody (Invitrogen, Carlsbad, CA, USA). The percentage of perfused vessels was calculated as the percentage of perfused vessels (yellow) compared to the amount of total vessels (green). Six random areas (1000×1000 µm) on each paraffin slide (five tumors per group) were analyzed for the calculation of vessel coverage, number of branched microvessels and vessel perfusion.

Vessel size was determined as the area [µm^2^] of CD34-positive vessels and quantified by automated measurement of minor and major vessel axis using CellProfiler software on entire tumor slides (5 tumors per group).

### Corneal angiogenesis model

The protocol was modified according to the method described by Rogers et al. (2007). Briefly, micropockets were prepared under a microscope at approximately 1 mm from the limbus to the top of the cornea using a surgical blade and sharp tweezers in the anesthetized mouse. The disc (Nylaflo®, Pall Corporation, Michigan) was implanted and the surface of the implantation area was smoothened. Discs were incubated in VEGF or in vehicle for at least 30 min. After 5 days, eyes were photographed and vascular response was measured. The assay was quantified by calculating the percentage of the area of new vessels per total area of the cornea.

### Mouse trachea assay

Female Balb/c mice were treated with 25 mg/kg ip control IgG, LC06, and LC08 once weekly x 14. Mice were perfused for 20 min, tracheas were removed and incised along the ventral midline, fixed in 4% PFA for 2 hr, and processed as whole mounts for immunohistochemistry, using rat α mouse CD31 (Pharmingen) as primary antibody. Signals were detected using goat α rat Alexa 488 (Invitrogen) as secondary antibody. Images (10x objective) were captured with a confocal microscope (Leica DMI6000B). Capillary branching points were counted in regions of 230×520 µm.

### Statistical analysis

All results were expressed as mean ± SEM. Differences between experimental groups were analyzed by unpaired Student's t-test. P values <0.05 were considered statistically significant.

## Results

### Anti-Ang-2 antibodies bind with high affinity and specifically to Ang-2

We have recently described two species cross-reactive Ang-2 antibodies LC06 and LC08 that were obtained from human antibody libraries by panning with Ang-2 and competitive elution with Tie2-Fc protein [22]. Table S1 summarizes the binding affinities as well as the interference with the Tie-2-Angiopoietin interaction in biochemical assays. LC06 can be considered as Ang-2 selective and does not interfere with the Tie2-Ang-1 interaction, whereas LC08 recognizes Ang-1 with significant affinity and inhibits the Tie2-Ang-1 interaction as well. In order to overcome avidity effects of the antibodies we established a novel competition surface plasmon resonance (SPR) assay format that excludes avidity effects due to the multimeric nature of Angiopoietin (Figure S1). The SPR data summarized in Figure S2B and Table S1 confirm similar single digit 1 nM affinity of LC06 and LC08 for human Ang-2 and a more than 1000-fold reduced affinity of LC06 for human Ang-2, whereas LC08 binds Ang-1 with ca. 10-fold lower 12 nM affinity. The results were confirmed in 2 independent experiments.

### Neutralization of ANGPT1/2-Tie2 interaction on cells results in inhibition of Tie2 phosphorylation

We have previously reported that LC06 and LC08 can interfere with the Ang-2-Tie2 and Ang-1-Tie2 interaction in biochemical interaction ELISAs [22]. In order to study the effects of Ang-2 and the respective antibodies on Tie2 downstream signaling we have generated a HEK293 clone stably transfected with human Tie2. Stimulation with Ang-2 or Ang-1 results in phosphorylation of Tie2 in these cells (data not shown). Both antibodies LC06 and LC08 were able to inhibit the binding of human Ang-2 to Tie2 on HEK293 cells in a single digit nM range (Table S2). Subsequently the ability of the identified antibodies to interfere with Ang-2- and Ang-1-mediated Tie2 phosphorylation was examined. While both antibodies LC06 and LC08 showed a dose-dependent interference with Ang-2 stimulated Tie2 phosphorylation with comparable IC50 values in the single digit nM range (Table S2), only LC08 interfered with Ang-1 stimulated Tie2 phosphorylation confirming the lower selectivity of LC08 for Ang-2 over Ang-1 compared to LC06.

### LC06 and LC08 show similar anti-tumor activity in human xenograft tumor models

**Figure 1:**
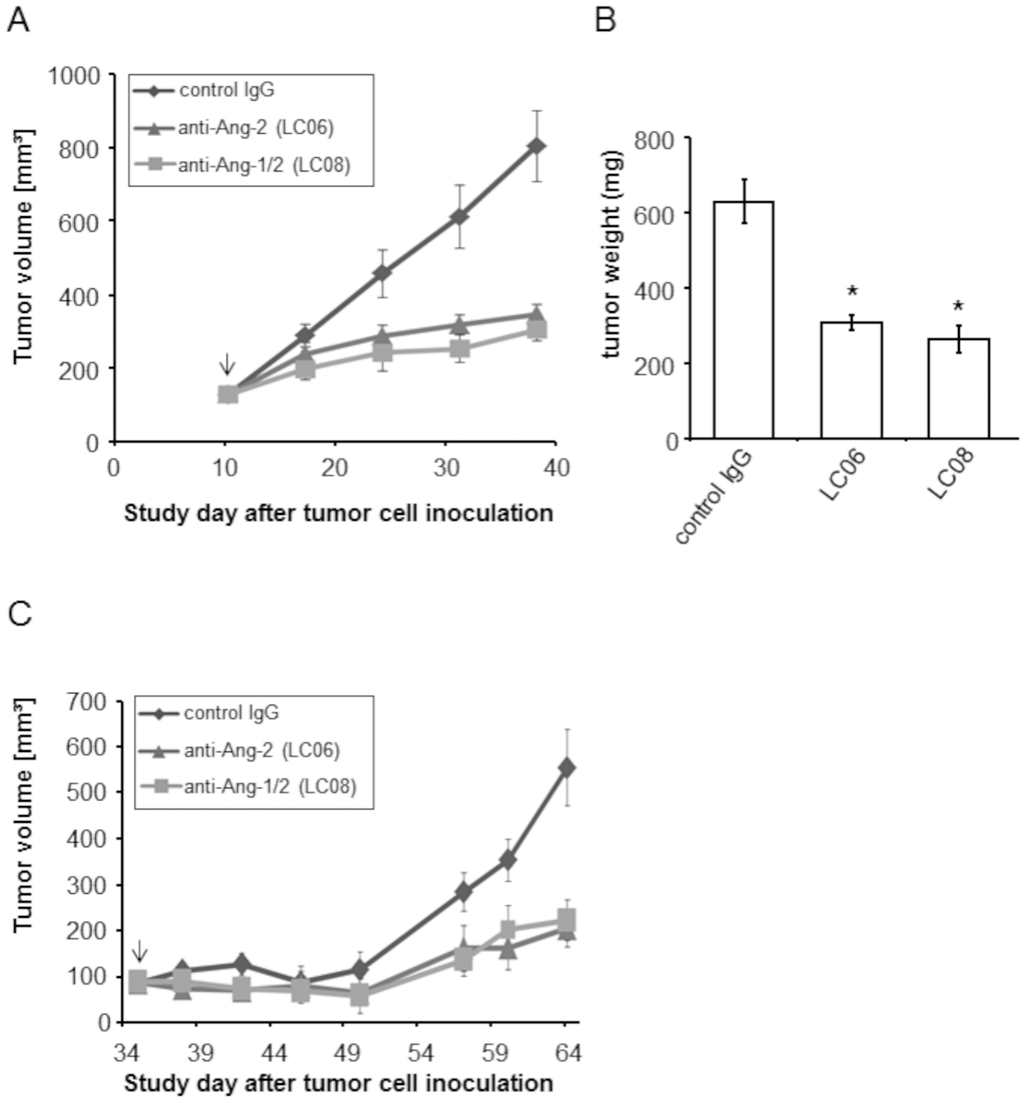
Anti-tumor activity of LC06 and LC08 in s.c. Colo205 and orthotopic KPL-4 xenograft tumors. (**A**) Colo205 tumor (mean 100 mm^3^) growth curves in female SCID beige mice receiving LC06 and LC08 (10 mg/kg) once weekly i.p. (n = 10, *p<0.0001, Student's t-test). (**B**) Total Colo205 tumor weight in tumor bearing mice treated with LC06 and LC08 (n = 10, *p<0.0001 compared to control, Student's t-test). (**C**) KPL-4 orthotopic tumor (mean 90 mm^3^) growth curves in mice receiving LC06 and LC08 (10 mg/kg) once weekly i.p. (n = 10, *p<0.05 compared to control, Student's t-test). Error bars represent ± SEM. Arrows indicate start of treatment. The results were confirmed in 3 independent experiments.

Up-regulation of host derived Ang-2 has been shown in many different tumors [23], [24]. To demonstrate the *in vivo* anti-tumor activity of LC06 and LC08 we studied the growth of established subcutaneous colorectal Colo205 and orthotopic mammary KLP-4 tumor xenografts in SCID beige mice under treatment. Colo205 tumor xenografts are an established model for anti-Ang-2 treatment [15], [25]. KPL-4 breast cancer cells were shown *in vitro* to express high levels of human Ang-2 (data not shown). LC06 and LC08 significantly inhibited tumor growth in both xenograft tumors (70–80% tumor growth inhibition; Figure 1A and 1B). LC06 inhibited the growth of s.c. Colo205 tumors by 70% (p<0.05) and the growth of orthotopic KPL-4 tumors by approx. 80% (p<0.05) compared to vehicle-treated controls. Tumor weight determined at the end of the study (day 38 for Colo205 and day 64 for KPL-4) was strongly reduced in Colo205 (Figure 1A) and KPL-4 (data not shown) bearing mice and correlated with TGI. The results were confirmed in 3 independent experiments and are in line with previously published results using LC06 and LC08 in Colo205 [22]. Antibody related toxicity was not observed for Colo205 and KPL-4 tumor bearing mice as shown by lack of changes in body weights (Figure S3A and S3B). The antibodies also did not cause any visual organ changes upon inspection. A more detailed histological analysis will be conducted in subsequent studies.

### Effect of LC06 and LC08 on microvessel density and vessel architecture

**Figure 2:**
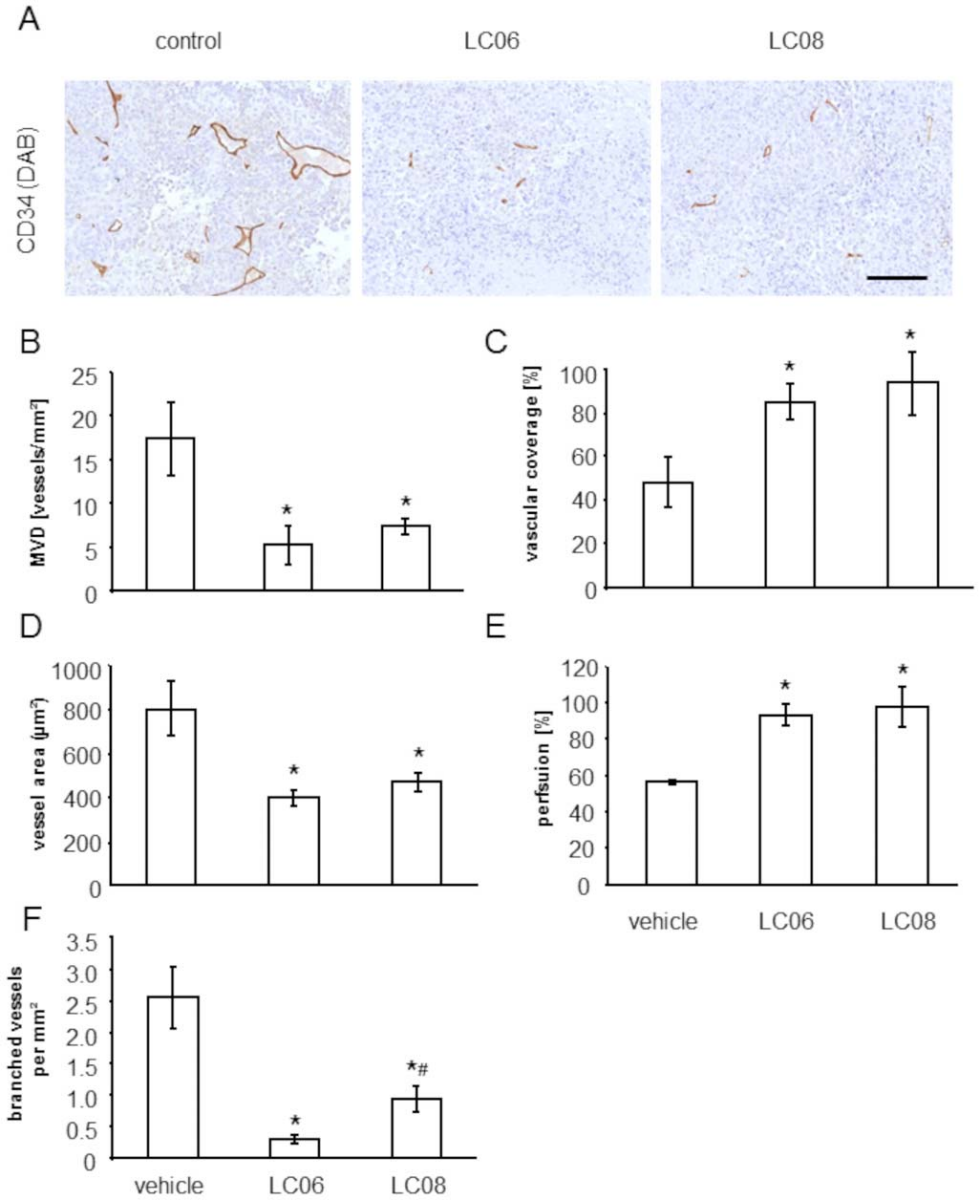
Effect of LC06 and LC08 treatment on MVD (A and B), vascular coverage (C), vessel area (D), perfusion (E) and vessel branching (F) in Colo205 tumors. (**A**) Representative pictures of CD34-stained Colo205 tumors (mean 800 mm^3^). (**B**) Quantitative analysis of tumor microvessel density (MVD). MVD was quantified in CD34-stained whole tumor slides. LC06 and LC08 treatment led to a significant reduction of intratumoral MVD (n = 5, *p<0.05 compared to control, Student's t-test). (**C**) Quantification of vessel coverage calculated as the percentage of desmin-positive vessels in relation to CD34-positive endothelial cells staining in six random regions of 1000×1000 µm per tumor slide. LC06 and LC08 treated tumors showed increased vessel coverage by desmin positive pericytes (n = 5 mice per group, *p<0.05 compared to control, Student's t-test). (**D**) The average vessel area of intratumoral microvessels were significantly reduced in tumors treated with LC06 and LC08 (n = 5, *p<0.05 compared to control, Student's t-test). (**E**) Perfusion was assessed based on analysis of TRITC-lectin perfusion and CD34-positive staining in six random regions of 1000×1000 µm per tumor slide. Almost all remaining intratumoral microvessels were perfused in LC06 (93%) and LC08 (97%) treated tumors compared to control (56%) (n = 5 mice per group, *p<0.01). (**F**) Number of branched intratumoral microvessels was counted in six random regions of 1000×1000 µm per tumor slide and calculated per mm^2^ and was significantly reduced in tumors treated with LC06 and LC08. LC06 treatment resulted in stronger inhibition of vessel branching compared to pan-Ang-2/-1 treatment via LC08 (n = 5, *p<0.01 compared to control; ^#^p<0.01 compared to LC08, Student's t-test). Results are expressed as mean ± SEM. Scale bars represent 500 µm.

**Figure 3:**
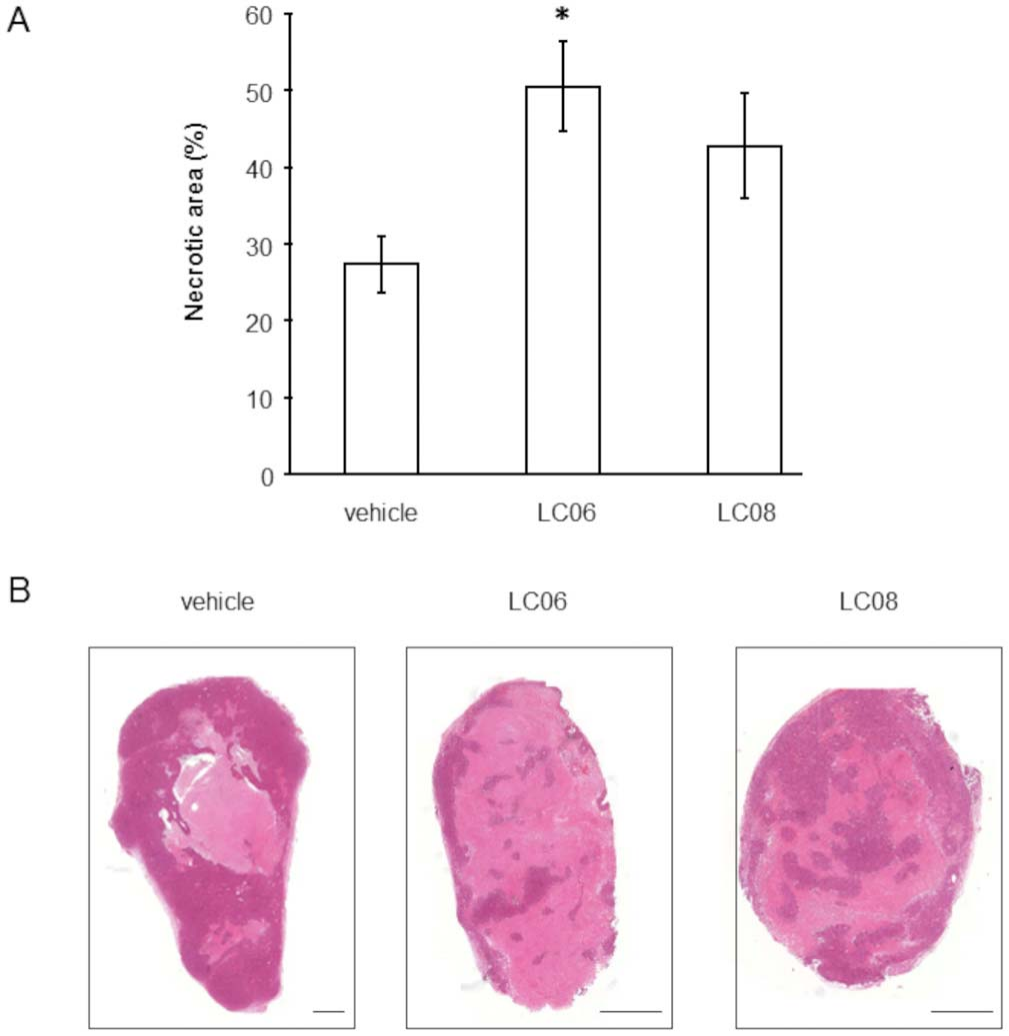
Tumor necrosis in LC06 and LC08 treated Colo205 tumors. (**A**) Quantitative image analysis shows 27% of necrotic area in vehicle treated, 51% in LC06 treated and 43% in LC08 treated tumors (n = 5, *p<0.05 compared to control, Student's t-test). Results are expressed as mean ± SEM. Two independent experiments were performed to confirm the results. (**B**) Representative mosaic images (10x) of vehicle, LC06 or LC08 treatments. Scale bar represents 1.3 mm.

Ang-1/Tie2 signaling has previously been shown to affect vessel architecture by affecting microvessel density (MVD) [14] and by decreasing vessel diameter and length [26]–[28]. To further elucidate the mechanism of action behind the observed tumor growth inhibition previously described for LC06 and LC08, we examined if this effect was related to alterations of the vessel architecture and the function of the intratumoral microvessels. MVD was decreased in tumors treated with LC06 and LC08 in the same range compared to vehicle-treated tumors (Figure 2A and 2B). Given the predominant role of the Ang/Tie system in regulating mural cell recruitment and vessel maturation [3], [29]–[32], we next analyzed mural cell recruitment by quantification of vascular coverage (desmin-positive cells in relation to CD34 positive vessels and NG2-positive cells in relation to CD31 positive vessels). The analysis demonstrates that the percentage of desmin- and NG2-positive mural cells associated to endothelial cells was significantly higher in LC06 and LC08 treated tumors compared to vehicle treated control (Figure 2C and Figure S4A and S4B), supporting the hypothesis of a vessel maturation phenotype. Correspondingly, pericyte coverage has been shown to alter the plasticity window of remodeling neovessels [33]. Thus, based on the observed pericyte recruitment in the tumors, we analyzed the vessel architecture in more detail. The average vessel area was smaller in tumors treated with LC06 and LC08 (Figure 2D). These changes in vessel area were associated with an increase in vessel perfusion in the LC06 and LC08 treated groups as assessed by TRITC-lectin perfusion (Figure 2E and Figure S5). We also discovered that the selective inhibition of Ang-2 with LC06 led to a stronger inhibition of vessel branching compared to the pan-Ang-2/-1 inhibition as mediated by LC08 (Figure 2F). Furthermore, tumor cell necrosis was significantly increased in mice treated with LC06 compared to unselective Ang-1/Ang-2 treatment by LC08 (Figure 3A and 3B). As anti-angiogenic drugs were shown to increase hypoxic regions in the tumor [34], we analyzed hypoxia in Colo205 tumors. Hypoxia analyzed at two different time points (day 34 and the end of the study, day 42) showed no differences in treatment compared to control groups (data not shown). Collectively, these findings suggest that the increased pericyte recruitment in treated tumors reflect a vascular remodeling phenotype with alterations in vessel size, branching and perfusion. Additionally, our findings of reduced MVD and tumor cell necrosis, but no increase in tumor hypoxia suggests that tumor vessel functionality may be restored under anti-Ang-2 therapy. The results were confirmed in two independent experiments.

### Effect of LC06 and LC08 on disseminated tumor cells

**Figure 4:**
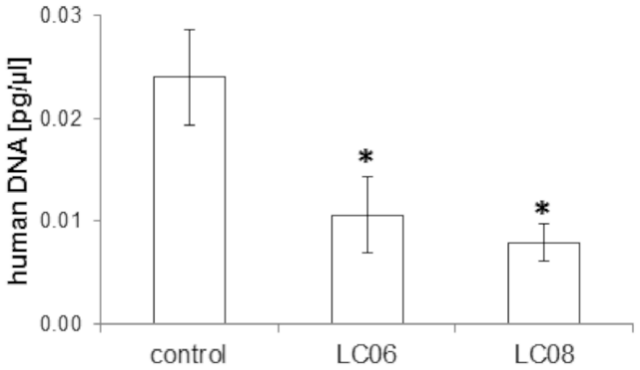
Tumor cell dissemination to the lungs in orthotopic KPL-4 xenograft tumors after LC06 and LC08 treatment. Treatment with LC06 and LC08 resulted in a significant reduction of disseminated tumor cells to the lungs (n = 10, *p<0.05 compared to control, Student's t-test). The results were confirmed in two independent experiments. Results are expressed as mean ± SEM.

It was previously reported that Ang-2 inhibition results in anti-metastatic activity in a spontaneous mammary carcinoma model [35]. Controversially, the effect of anti-VEGF/R inhibitors on metastasis formation has been a matter of debate as data demonstrate that inhibition of VEGFR-2 with monoclonal antibodies or small molecules lead to an increase in metastases and more invasive tumor cell phenotypes [36]–[38]. Interestingly, tumor cell dissemination to the lungs of mice bearing orthotopic KPL-4 tumors (Figure 1B) analyzed at the end of the study (day 91) was significantly inhibited in LC06 and LC08 treated animals (Figure 4). Quantification of human DNA in the mouse lungs was achieved by Alu-PCR analysis and the results were confirmed in two independent experiments.

### Effect of LC06 and LC08 on quiescent vessels

**Figure 5:**
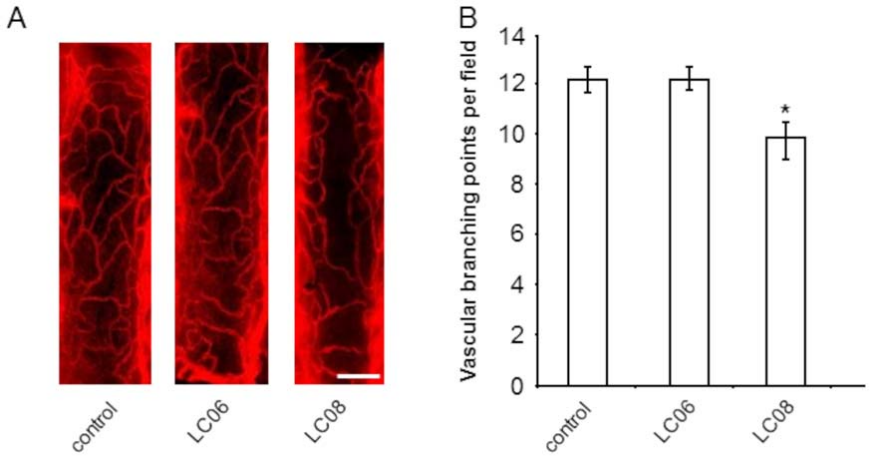
Effect of LC06 and LC08 on quiescent vessels. (**A**) Representative immunofluorescent pictures of CD31 stained tracheal whole mount sections of mice treated with control IgG_1_, LC06 and LC08. (**B**) Quantification of capillary branching points (in five random regions of 230×520 µm in each mouse whole mount trachea) reveals that pan-Ang-1/Ang-2 inhibition but not selective anti-Ang-2 treatment induced tracheal vessel regression (n = 3–5; *p<0.05 compared to control, Student's t-test). The results were confirmed in two independent experiments. Results are expressed as mean ± SEM. Scale bar represents 100 µm.

Next, we sought to analyze the effect of Ang-2 selective and non-selective antibodies on healthy vessels in the mouse trachea. Selective Ang-2 inhibition did not affect healthy vessels compared to the control group (Figure 5A and 5B) while anti-Ang-1/-2 treatment resulted in regression of healthy vessels in the mouse trachea (Figure 5A and 5B). These results imply a key difference between selective Ang-2 and unselective Ang-1/Ang-2 inhibition with potential implications for safety. The results were confirmed in two independent experiments.

### Activity of LC06 in the corneal angiogenesis assay

**Figure 6:**
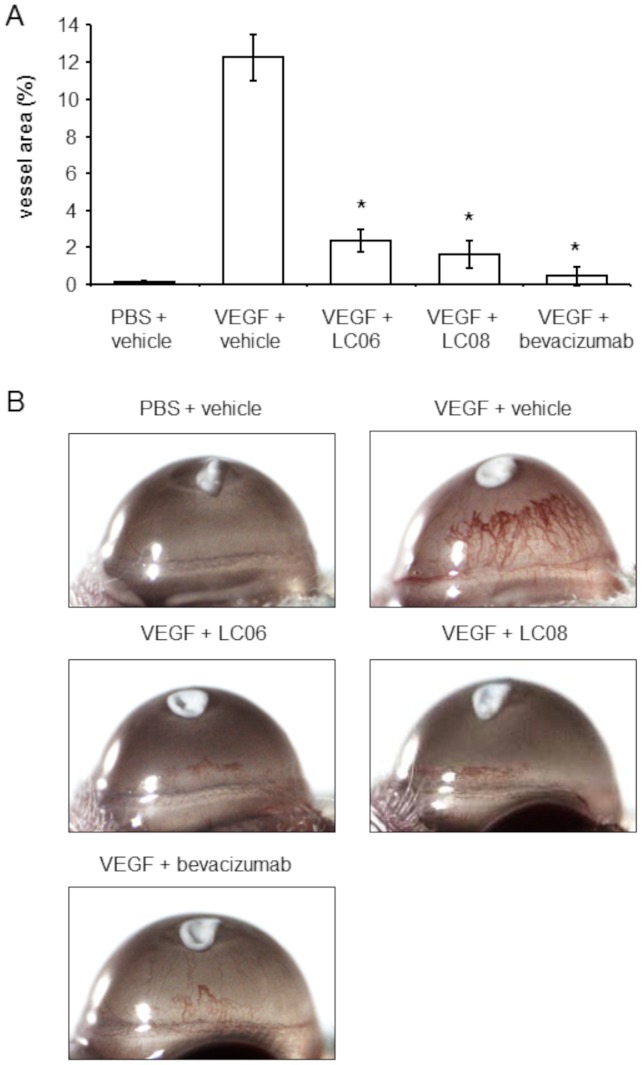
Effect of LC06 and LC08 treatment on VEGF-induced corneal angiogenesis. (**A**) Angiogenesis was induced by implanting VEGF (300 ng) or vehicle soaked nylaflo discs into the cornea of BALB/c mice. PBS control did not induce vessel outgrowth from the limbus to the disc compared to VEGF positive control. LC06, LC08 and bevacizumab were injected with 10 mg/kg i.v. at the day of disc implantation. Treatment with both anti-Ang-2 antibodies significantly inhibited neoangiogenesis in a comparable range as bevacizumab (n = 5, *p<0.001 compared to VEGF control, Student's t-test). (**B**) Representative pictures of vehicle, LC06 and LC08 treated mice. The results were confirmed in 3 additional independent experiments. Results are expressed as mean ± SEM.

Corneal angiogenesis assays imply that both Ang-1 and Ang-2 have similar effects, acting synergistically with VEGF to promote growth of new blood vessels. Inhibition of Ang-2 has been shown to prevent VEGF-induced tumor angiogenesis in a rat cornea pocket assay [25] and tumor cell-induced angiogenesis in a matrigel plug assay [14]. The possibility for a dose-dependent endothelial response was raised by the observation that *in vitro* at high concentrations Ang-2 can be pro-angiogenic [8]. At high concentrations, Ang-2 acts as an apoptosis survival factor for endothelial cells during serum deprivation apoptosis through activation of Tie2 via PI-3 Kinase and Akt pathways [8]. Therefore, we analyzed the effect of Ang-2 inhibition by LC06 and LC08 on VEGF-induced angiogenesis in the mouse corneal angiogenesis assay. Our results demonstrate that systemic administration of LC06 and LC08 at a dose of 10 mg/kg very efficiently inhibited the outgrowth of vessels from the limbus towards the VEGF gradient (Figure 6A and 6B). Notably, blocking VEGF-induced angiogenesis with LC06 and LC08 was as efficient in inhibiting angiogenesis as bevacizumab. Taken together, our data support the conclusion that Ang-2 neutralization suppresses angiogenesis in a neoplastic and non-neoplastic setting. The results were confirmed in three independent experiments.

## Discussion

The Angiopoietins (Ang-1 and Ang-2) and their receptors Tie1 and Tie2 are involved in the formation and maturation of new vessels during embryogenesis and pathology [3], [11], [39]. Here we describe a novel fully human and species cross-reactive Ang-2 selective antibody, LC06, and a corresponding Ang-2/-1 cross-reactive antibody, LC08. In this respect, the antibodies mediate their mode of action by neutralizing either specifically Ang-2 (LC06), or Ang-2 as well as Ang-1 (LC08) at pharmacologically relevant levels achieved in animal models.

In clinical PhII studies of recurrent ovarian cancer, combination of Ang 1/2 cross-reactive molecule AMG386 and Paclitaxel resulted in prolonged progression free survival in a dose dependent manner (from 4.6 to 7.2 month for 10 mg/kg AMG386 plus paclitaxel) [40]. However, no additional anti-tumoral activity was observed in first line metastatic renal cancer in combination with Sorafenib [41]. The most common side effects of AMG386 were fatigue and peripheral edema [19].

Ang-1 is constitutively expressed by numerous cell types and controls the vascular quiescent endothelial cell phenotype in the adult [42]. In contrast, the contextually agonistic and antagonistic ligand Ang-2 is almost exclusively produced by endothelial cells (EC) and thereby controls vascular homeostasis through an autocrine loop mechanism [2], [3], [8], [9], [27]. Antagonistic functions of Ang-2 are demonstrated in Ang-2 overexpressing mice that show a similar phenotype as Ang-1 and Tie2-deficient mice [3]. Even though Ang-2 is thought to be the antagonist of Ang-1 *in vivo*, *in vitro* experiments show that both proteins can have similar functions dependent on ligand concentration, stimulation time, cell type, confluency and activation [7]–[9], [43]–[45]. In tumors (e.g., gastric, colon, prostate, breast, AML and brain carcinomas), Ang-2 in contrast to Ang-1 is up-regulated and strongly correlates with tumor angiogenesis and poor prognosis. [11]–[13]. Despite the prognostic relevance of Ang-2 in acute myeloid leukemia, Ang-1 was also shown to be released from primary AML cells contributing to leukemogenesis and chemosensitivity [46]. In the tumor context, our present study shows that the neutralization of both Angiopoietins with LC06 and LC08 results in a potent anti-tumoral efficacy in tumor xenografts and angiogenesis models as previously reported [14], [25], [29], [47]. Treatment with LC06 and LC08 significantly reduced tumor microvessel density (MVD). Furthermore, LC06 and LC08 treated mice showed a strong decrease in blood vessel formation in the corneal VEGF-induced angiogenesis assay to the same level as bevacizumab. In contrast to published results [48] we show that unselective Ang-1/Ang-2 neutralization results in similar anti-tumoral activity compared to selective Ang-2 targeting.

High levels of Ang-2 correlate with increased metastatic and invasive potential in breast cancer, malignant melanoma and lung cancer [49]–[51]. Ang-2 is also often expressed at the invasive fronts of human tumors, such as metastatic melanoma, invasive ductal breast carcinoma and glioma [49], [52], [53]. Preclinical data with inhibitors against Ang-2 strongly suggest that inhibiting Ang-2 will not only have an effect on tumor angiogenesis, but may also decrease the metastatic potential [15], [35]. Our data demonstrate that treatment of an orthotopic breast cancer xenograft with LC06 and LC08 reduced the dissemination of tumor cells to the lungs. The effect of LC06 and LC08 was very similar indicating that tumor cell dissemination is mostly triggered via Ang-2 in the tested models. Our results cannot be correlated with the complete process of metastasis, as the lungs in our study were not perfused before harvesting.

The signaling pathways of the Angiopoietins during vessel maturation are also incompletely understood. Tumors grown in Ang-2-deficient mice have been shown to have a more profound vessel maturation phenotype [30]. In line with these results, we can show that a specific inhibition of Ang-2 and a pan-Ang-2/-1 inhibition results in a stronger coverage of vessels with desmin positive cells. Furthermore, Ang-2 treated tumors had smaller vessels and showed less branching corresponding to the vessel diameter sensing role of Ang-1 and Tie2 [26], [27].

Anti-angiogenic therapies were shown to increase hypoxia in the tumor [34]. In the present study, tumor hypoxia was analyzed at two different time points (day 34 and at the end of the study, day 42). Despite detection of pronounced tumor necrosis, no differences regarding hypoxia were detectable in the treatment group compared to the control group. Selective Ang-2 inhibition further reduced vessel branches compared to a pan-Ang-2/-1 treatment indicating the important role of Ang-1 in vessel stabilization. Interestingly, vessel pericyte coverage is massively increased in Ang-1 overexpressing tumors, which argues for a stabilization of tumor vasculature [54]. In line with these data, we could demonstrate for the first time that healthy vessels in the mouse trachea were not affected by a selective anti-Ang-2 treatment in contrast to an unselective approach targeting Ang-1 and Ang-2. This is in support of the hypothesis that Ang-1 acts as a vessel maintenance factor. These data, together with the fact that Ang-2 can be found to be up-regulated in pathological conditions but does not have an essential physiological role in adults are supportive of a favorable safety profile of a selective anti-Ang-2 therapy.

Given the comparable efficacy and the essential role of Ang-1 in vessel maintenance we conclude that in contrast to Falcon et al. (32) the Ang-2 selective LC06 antibody may be the preferred therapeutic agent over the Ang-1 cross-reactive antibody LC08. Interfering with vessel maintenance by an unselective Ang-1/Ang-2 inhibition may lead to toxicology related complications such as edema observed in clinical trials [19].

The favorable safety profile of Ang-2 selective antibodies in combination with their anti-tumor, anti-metastatic and anti-angiogenic potential demonstrate a substantial benefit of Ang-2 targeting in oncology whereas the benefits of Ang-1 targeting remain debatable. An attractive scenario might be the combination of anti-Ang-2 therapies with other anti-angiogenic molecules to prevent or overcome escape mechanism and to further inhibit metastasis formation in combination therapy or in bispecific antibodies targeting Ang-2 and VEGF [55].

## Supporting Information

Figure S1 Binding of Ang-1 and Ang-2 to LC06 and LC08 measured by SPR. SPR data confirm similar high affinities of all tested antibodies targeting human Ang-2. LC08 in contrast to LC06 shows also significant binding towards human Ang-1. Two independent experiments were performed to confirm the results.
(TIF)

Figure S2 Setup of surface plasmon resonance. The antibody of interest (LC06 or LC08) was immobilized on the surface of a C1 SPR Chip. Fixed concentrations of hAng2 or hAng1 were pre-incubated together with increasing concentrations of the corresponding antibody and injected onto the flowcells.
(TIF)

Figure S3 Effect of LC06 and LC08 treatment on body weight of SCID beige mice bearing KPL-4 and Colo205 tumors. No significant toxicity was observed as demonstrated by the changes in body weights for SCID beige mice bearing KPL-4 (A) and Colo205 (B) tumors (n = 10). Arrows indicate start of treatment. The results were confirmed in two additional independent experiments.
(TIF)

Figure S4 Staining of desmin-positive and NG2-positive vessels. Representative images (20x) of vehicle, LC06 or LC08 treatment are shown. Association of desmin- (A) and NG2 (B) positive cells (green) with tumor vessels (red) is significantly increased after LC06 and LC08 treatment of Colo205 tumors. Colocalized staining (yellow) of pericytes (green) and endothelial cells (red) can be detected. Scale bar: 500 µm.
(TIF)

Figure S5 Staining of perfused vessels. Representative images (20x) of vehicle, LC06 or LC08 treatment are shown. Perfused vessels (red; i.v. injection of lectin-TRITC) appear yellow as they are superimposed on the CD34 staining (green). While treatment with LC06 and LC08 reduced microvessel density in Colo205 tumors it increased the percentage of lectin perfused vessels (yellow) compared to the total amount of remaining vessels (green). Scale bar: 500 µm.
(TIF)

Table S1 Cross-reactivity of LC06 and LC08 to murine, cynomolgus monkey and human Ang-2, and inhibition of Ang-1 and Ang-2 binding to Tie2 by LC06 and LC08 determined by ELISA and SPR. The binding of Ang-2 antibodies LC06 and LC08 to human Ang-1 and human Ang-2 was determined in an ELISA and on SPR. LC06 binding to human Ang-2 was determined with an EC50 value of 56.1 pM whereas the binding to human Ang-1 was >13333 pM. The binding of LC08 to human Ang-2 was determined with an EC50 value of 73.3 pM whereas the binding to human Ang-1 was 2.2 nM. LC06 and LC08 bind with high affinity to cynomolgus (EC50 of 40.6 pM for LC06 and 64.2 pM for LC08) and murine Ang-2 (EC50 of 38.8 pM for LC06 and 88.6 pM for LC08). The SPR data confirm similar single digit nM affinity of all tested antibodies targeting human Ang-2. The ELISA results were calculated from two independent experiments (n value  = 3).
(TIF)

Table S2 Binding of LC06 and LC08 in Tie2 ELISA and FACS analysis. Blocking of human Ang-1/Ang-2 to human Tie2 interaction was shown by receptor interaction ELISA. LC06 was found to bind Ang-2 with an IC50 value of 79 pM whereas the ability of the antibody to bind Ang-1 was determined with an IC50 above 50000 pM (detection limit). LC08 was found to bind Ang-2 with an IC50 value of 104 pM whereas the ability of the antibody to bind Ang-1 was determined with an IC50 of 4368 pM. The ELISA results were calculated from two independent experiments (n value  = 3). FACS analysis confirmed the comparable binding of LC06 (1 nM) and LC08 (1.3 nM) to Ang-2. The FACS results were confirmed in a second independent experiment.
(TIF)
