# Supplementary figures and images for: A Novel Angiopoietin-2 Selective Fully Human Antibody with Potent Anti-Tumoral and Anti-Angiogenic Efficacy and Superior Side Effect Profile Compared to Pan-Angiopoietin-1/-2 Inhibitors
Source: PLoS One. 2013 Feb 6;8(2):e54923. doi: 10.1371/journal.pone.0054923 (PMC3566157; doi:10.1371/journal.pone.0054923)

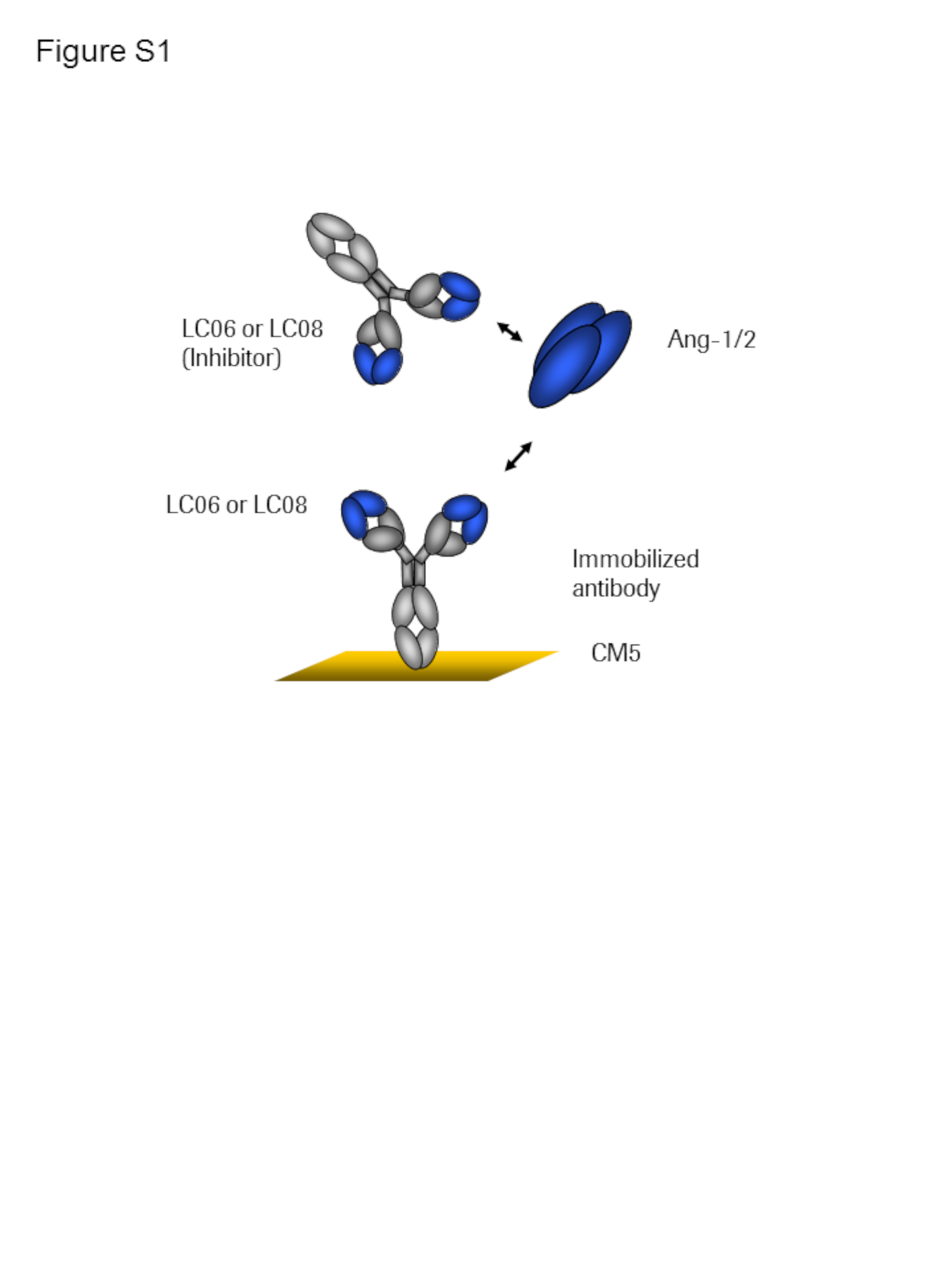

Supplement: Figure S1 — Binding of Ang-1 and Ang-2 to LC06 and LC08 measured by SPR. SPR data confirm similar high affinities of all tested antibodies targeting human Ang-2. LC08 in contrast to LC06 shows also significant binding towards human Ang-1. Two independent experiments were performed to confirm the results. (TIF) [file pone.0054923.s001.tif]

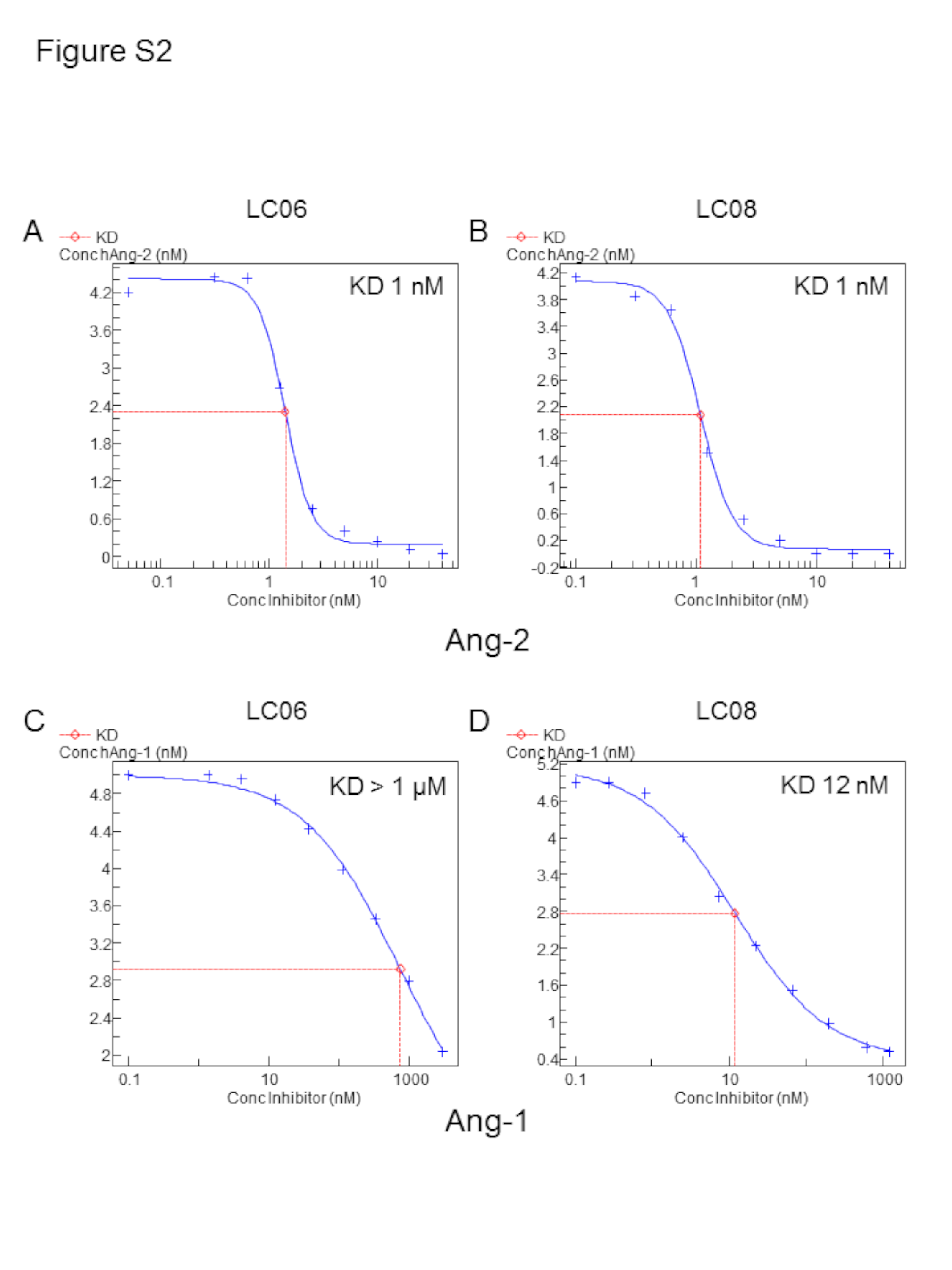

Supplement: Figure S2 — Setup of surface plasmon resonance. The antibody of interest (LC06 or LC08) was immobilized on the surface of a C1 SPR Chip. Fixed concentrations of hAng2 or hAng1 were pre-incubated together with increasing concentrations of the corresponding antibody and injected onto the flowcells. (TIF) [file pone.0054923.s002.tif]

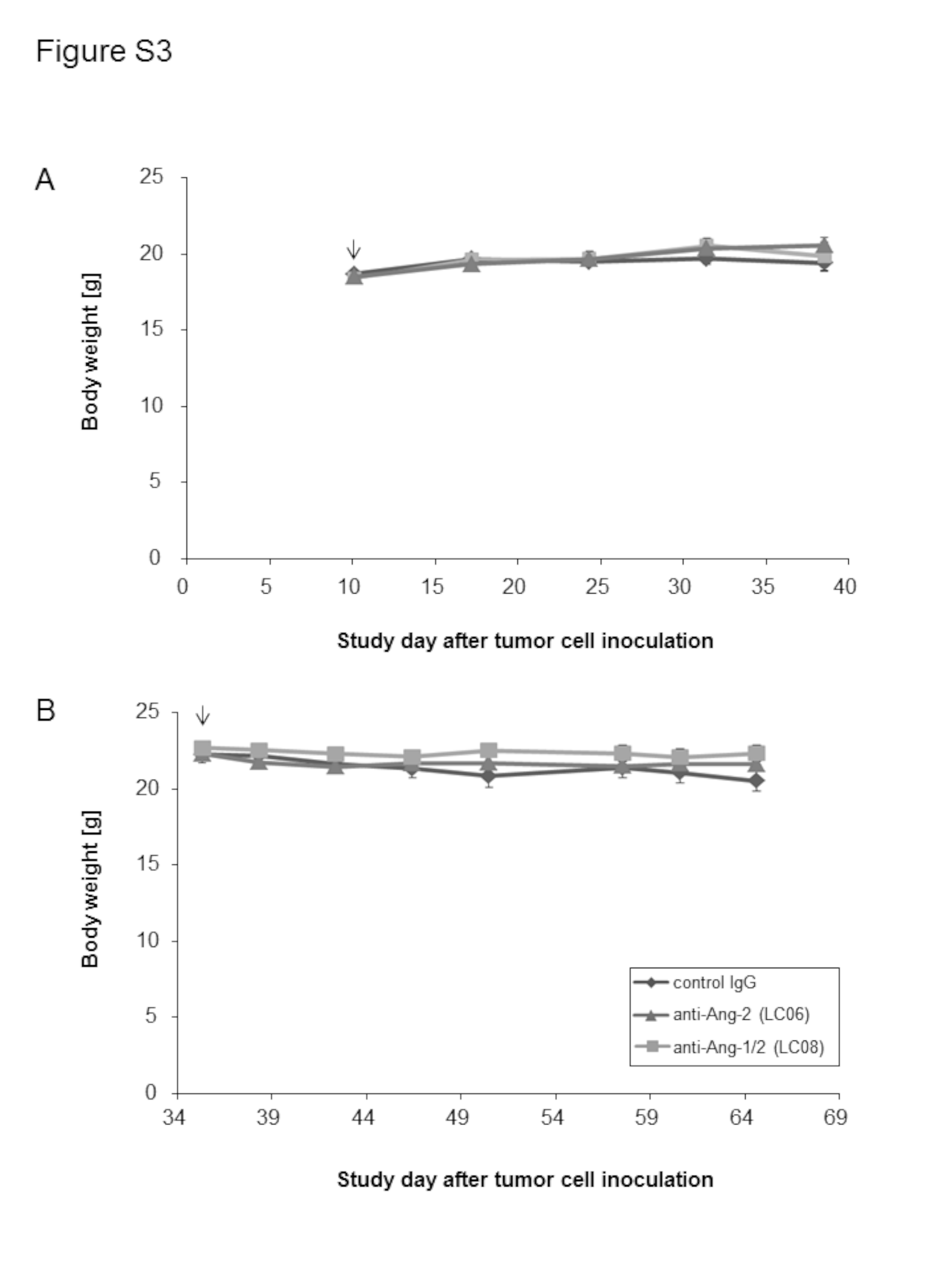

Supplement: Figure S3 — Effect of LC06 and LC08 treatment on body weight of SCID beige mice bearing KPL-4 and Colo205 tumors. No significant toxicity was observed as demonstrated by the changes in body weights for SCID beige mice bearing KPL-4 (A) and Colo205 (B) tumors (n = 10). Arrows indicate start of treatment. The results were confirmed in two additional independent experiments. (TIF) [file pone.0054923.s003.tif]

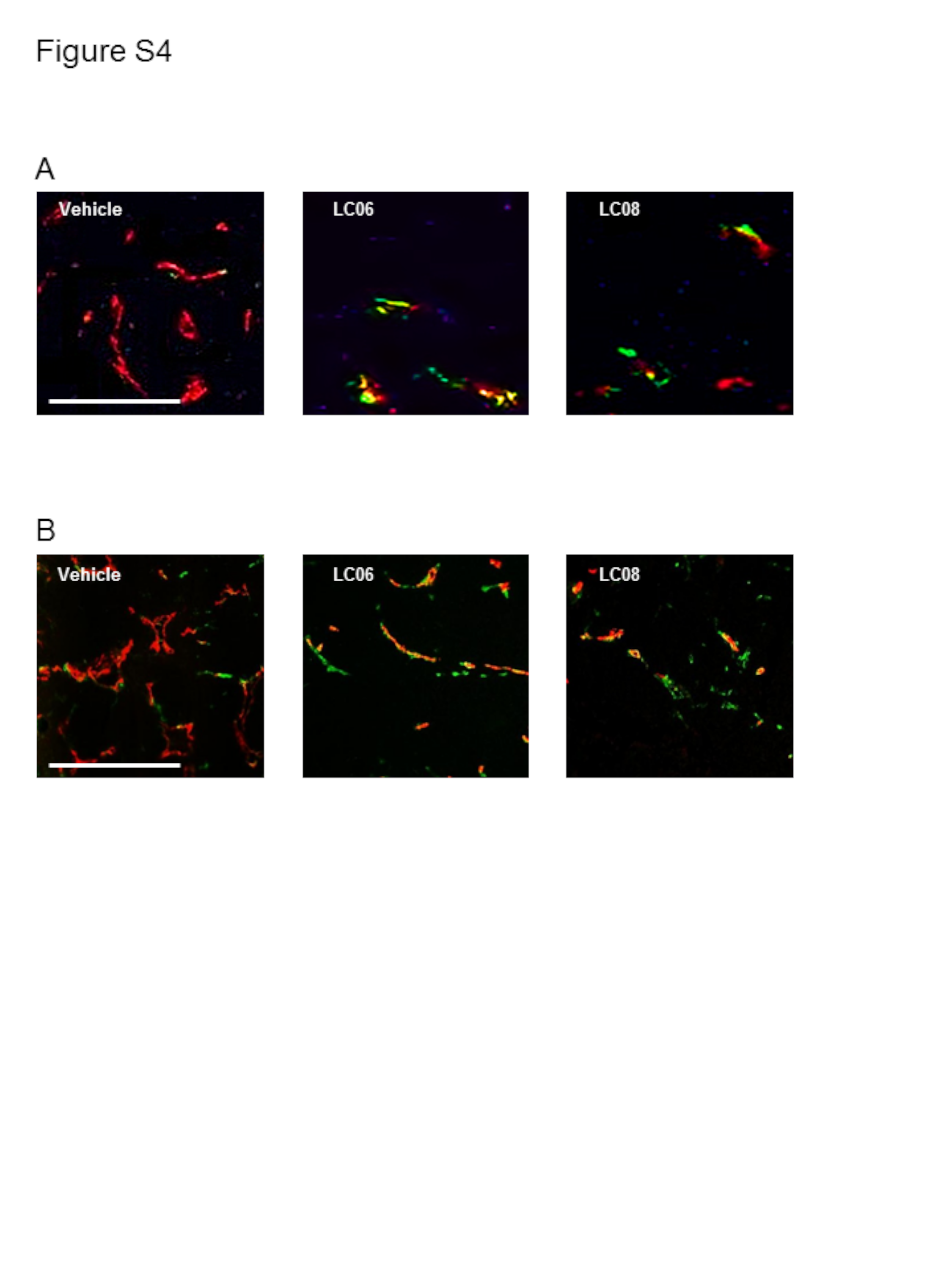

Supplement: Figure S4 — Staining of desmin-positive and NG2-positive vessels. Representative images (20x) of vehicle, LC06 or LC08 treatment are shown. Association of desmin- (A) and NG2 (B) positive cells (green) with tumor vessels (red) is significantly increased after LC06 and LC08 treatment of Colo205 tumors. Colocalized staining (yellow) of pericytes (green) and endothelial cells (red) can be detected. Scale bar: 500 µm. (TIF) [file pone.0054923.s004.tif]

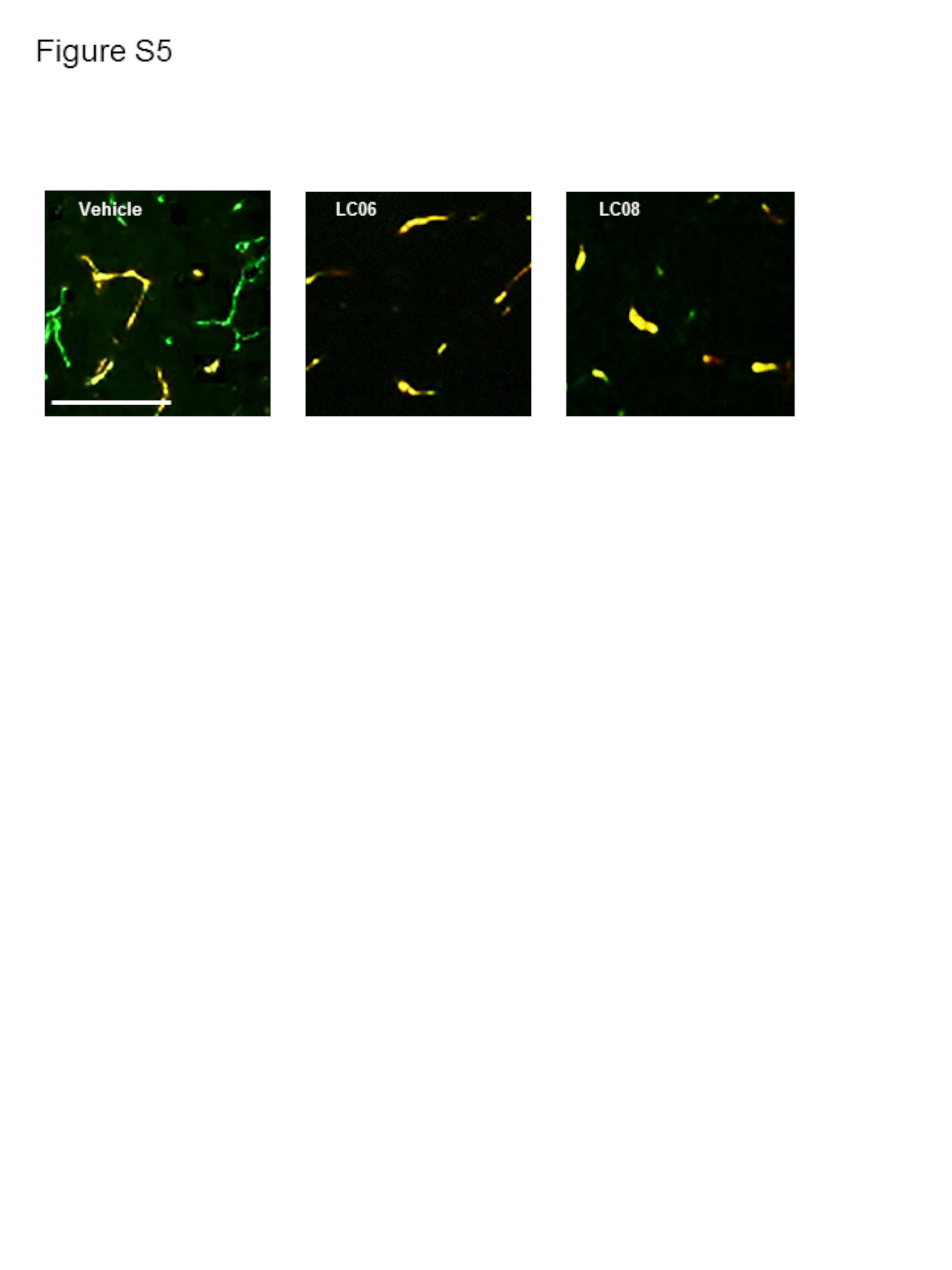

Supplement: Figure S5 — Staining of perfused vessels. Representative images (20x) of vehicle, LC06 or LC08 treatment are shown. Perfused vessels (red; i.v. injection of lectin-TRITC) appear yellow as they are superimposed on the CD34 staining (green). While treatment with LC06 and LC08 reduced microvessel density in Colo205 tumors it increased the percentage of lectin perfused vessels (yellow) compared to the total amount of remaining vessels (green). Scale bar: 500 µm. (TIF) [file pone.0054923.s005.tif]

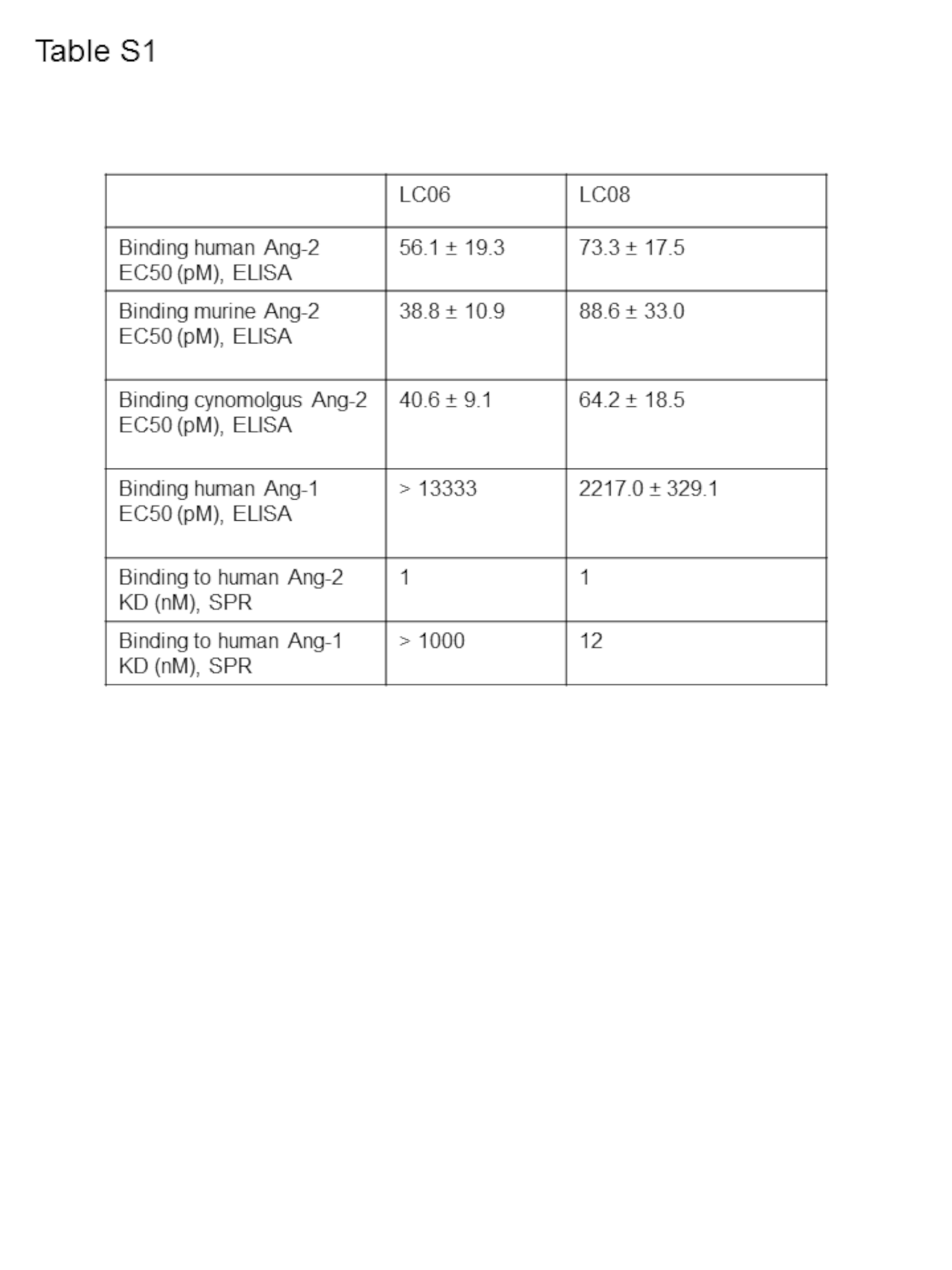

Supplement: Table S1 — Cross-reactivity of LC06 and LC08 to murine, cynomolgus monkey and human Ang-2, and inhibition of Ang-1 and Ang-2 binding to Tie2 by LC06 and LC08 determined by ELISA and SPR. The binding of Ang-2 antibodies LC06 and LC08 to human Ang-1 and human Ang-2 was determined in an ELISA and on SPR. LC06 binding to human Ang-2 was determined with an EC50 value of 56.1 pM whereas the binding to human Ang-1 was >13333 pM. The binding of LC08 to human Ang-2 was determined with an EC50 value of 73.3 pM whereas the binding to human Ang-1 was 2.2 nM. LC06 and LC08 bind with high affinity to cynomolgus (EC50 of 40.6 pM for LC06 and 64.2 pM for LC08) and murine Ang-2 (EC50 of 38.8 pM for LC06 and 88.6 pM for LC08). The SPR data confirm similar single digit nM affinity of all tested antibodies targeting human Ang-2. The ELISA results were calculated from two independent experiments (n value = 3). (TIF) [file pone.0054923.s006.tif]

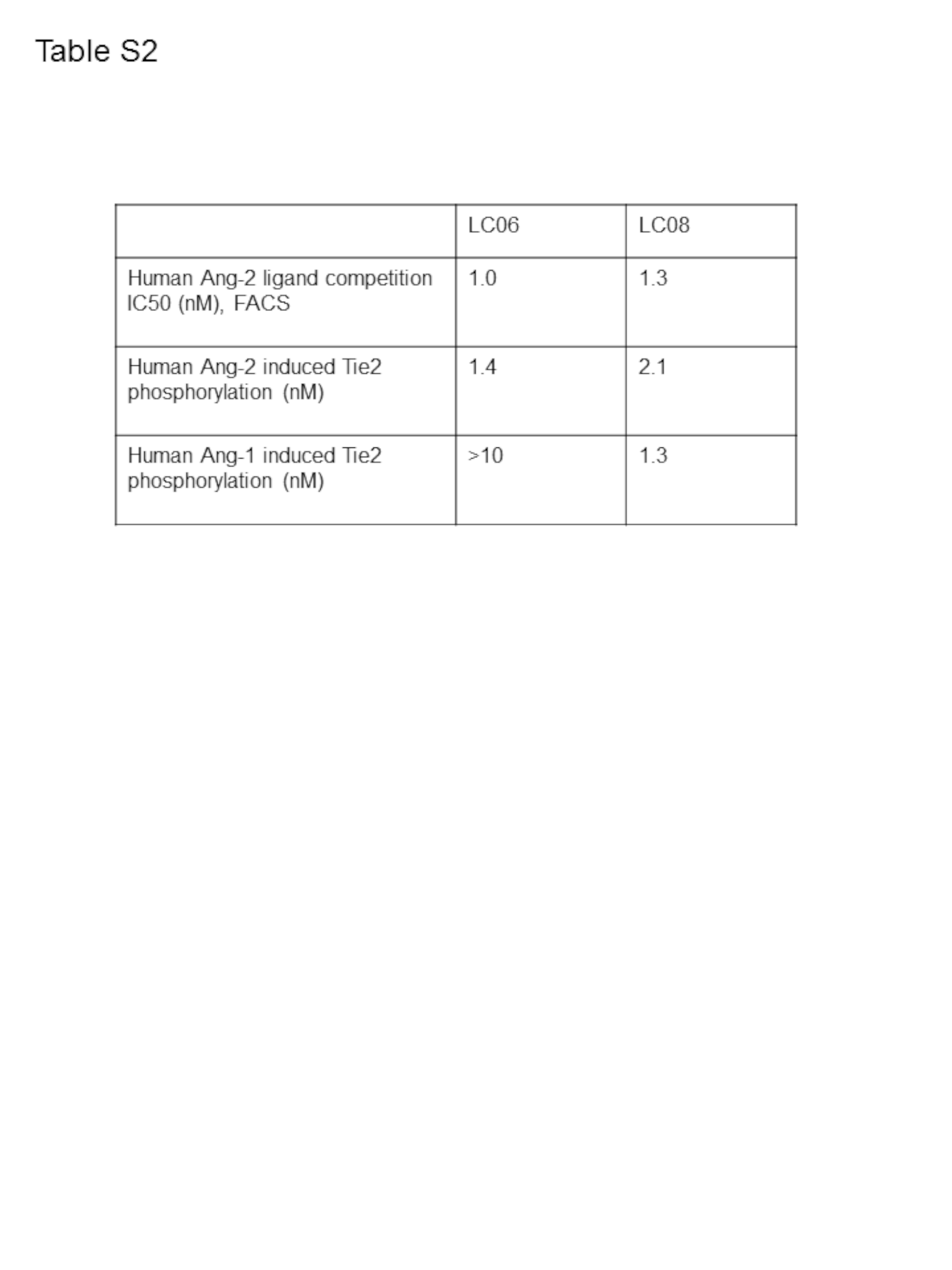

Supplement: Table S2 — Binding of LC06 and LC08 in Tie2 ELISA and FACS analysis. Blocking of human Ang-1/Ang-2 to human Tie2 interaction was shown by receptor interaction ELISA. LC06 was found to bind Ang-2 with an IC50 value of 79 pM whereas the ability of the antibody to bind Ang-1 was determined with an IC50 above 50000 pM (detection limit). LC08 was found to bind Ang-2 with an IC50 value of 104 pM whereas the ability of the antibody to bind Ang-1 was determined with an IC50 of 4368 pM. The ELISA results were calculated from two independent experiments (n value = 3). FACS analysis confirmed the comparable binding of LC06 (1 nM) and LC08 (1.3 nM) to Ang-2. The FACS results were confirmed in a second independent experiment. (TIF) [file pone.0054923.s007.tif]
